# Effect of post-weld heat-treatment and solid-state thermomechanical treatment on the properties of the AA6082 MIG welded joints

**DOI:** 10.1038/s41598-024-53795-6

**Published:** 2024-02-22

**Authors:** Hossam Hemdan El-Fahhar, Elshafey Ahmed Gadallah, Mohamed I. A. Habba, Mohamed M. El-Sayed Seleman, Mohamed M. Z. Ahmed, Abdelkarim Yousif Mohamed, Ramy A. Fouad

**Affiliations:** 1 Mechanical Production Department, Faculty of Technology and Education, Suez University, Suez, 43221 Egypt; 2 Metallurgical and Materials Engineering Department, Faculty of Petroleum and Mining Engineering, Suez University, Suez, 43221 Egypt; 3 Mechanical Engineering Department, College of Engineering at Al Kharj, Prince Sattam Bin Abdulaziz University, 16273 Al Kharj, Saudi Arabia

**Keywords:** AA 6082-T6, Metal inert gas, Thermomechanical treatment, Heat treatment, Mechanical properties, Mechanical engineering, Characterization and analytical techniques

## Abstract

Post-weld heat treatment (PWHT) and solid-state thermomechanical treatment (TMT) via friction stir processing (FSP) have been shown to enhance the mechanical properties of aluminum alloys. The current work investigates the effects of PWHT and TMT on the microstructure and mechanical performance of AA6082-T6 welded butt joints welded using the MIG process. The 5 mm thick AA 6082-T6 plates were joined in butt configuration using MIG welding with ER 5356 filler wire, 120 A current, 0.3 mm/s weld speed, and argon shielding gas at 15 L/min flow rate. PWHT was performed on the MIG welds per the T6 temper procedure. TMT was implemented via FSP using a pinless tool rotating at 800 rpm and traversing speed at 200 mm/min with a 3° tilt angle. Microstructural analysis, hardness mapping, tensile testing, and fracture surface evaluation were utilized to characterize the as-welded, PWHT, and TMT samples. The results demonstrate that both PWHT and TMT significantly refine and homogenize the microstructures of the welded joints. However, the TMT samples displayed superior hardness and tensile strength compared to the as-welded and PWHT conditions. The TMT-processed welds achieved approximately 99% joint efficiency versus only 69% and 85% for the as-welded and PWHT samples. In summary, PWHT and especially TMT via FSP are effective at enhancing the mechanical properties of MIG welded AA6082-T6.

## Introduction

Aluminum alloys in 6xxx are of great interest in the automotive industry. Automotive manufacturers demand lightweight materials such as aluminum alloys for structural components to reduce fuel consumption and weight while enhancing overall performance^1,2^. The 6xxx series of aluminum alloys show a high possibility for weight reduction in automotive and other vehicle industries, especially AA 6082 aluminum alloy^2,3^. AA 6082 alloy has medium to high strength and corrosion resistance and is strengthened by heat treatment, such as T6 temper conditions^4,5^. The increased strength-to-weight ratio after the heat treatment processing opens the applicability of this aluminum alloy in the automotive industry to fabricate automobile body components as potential alternative materials rather than steel^6^. However, their poor weldability and difficulty in producing high strength, fracture resistance, and fatigue welds are the main hindrances when this alloy is considered an alternative for steel joints during the fabrication of automobile body parts. The Metal Inert Gas (MIG) technique was mainly used in welding aluminum alloys, producing welds with good appearance and quality^7,8^. Welding of Al alloys is an active area for weight reduction in the automotive industry^9^. The microstructure and mechanical properties of the welded zone material in terms of hardness and tensile properties will differ from the material properties before welding^10^. A heat treatment process may be used to obtain the desired weld material properties. Heat treatment concerns different heating and cooling to cause changes in a material's microstructure, which affects its mechanical properties^11^. Several studies have investigated the effect of post-weld heat treatment (PWHT) on welds using different fusion welding techniques^12,13^. Akhter et al.^14^ studied the influence of pre and post-weld T6 heat treatment on the laser-welded A 356 cast aluminum alloy. They remarked that the hardness of the post-weld heat treatment (T6) joint is higher than the pre-weld heat treatment joint and the die-cast material. Nikseresht et al.^15^ investigate the effect of the heat treatment process on the AA 6061 gas tungsten arc welds. They remarked that the microstructure morphology changed from irregular to an almost spherical shape. Friction stir processing (FSP) is a modern solid state thermomechanical treatment (TMT) process derived from the established Friction Stir Welding (FSW) joining technique^16,17^. In the FSP process, a rotational tool (with or without a pin) is gradually introduced into the processing plate. This initiates significant plastic deformation and dynamic recrystallization in the material, driven by the tools mechanical stirring action^18–20^. Diverging from FSW, and FSP aims to enhance the structure of the stirring zone and improve material properties. Simultaneously, it has the capability to alter the surface of large workpieces by displacing the stirring tool^21,22^. Key advantages of FSP encompass its rapid and compact processing, achieving material uniformity and densification within stirring zones^23^. Moreover, modifying the stirring tools characteristics adjusts the processing zones shape and size. FSP directly processed the material surface, ensuring the modified layer remains integrated with the base material^24,25^. Also, FSP is characterized by its simplicity, efficiency, energy efficiency, and eco-friendly attributes, presenting extensive prospects for diverse applications^26^. The plastic deformation and rheology experienced by the materials in the processing zone make FSP suitable for many applications like processing welded material, surface of materials, and producing the surface composite^27^. During fusion welding processes, the intense heating and subsequent rapid cooling lead to the formation of coarse grains and segregation of alloying elements in the fusion zone and heat-affected zone^28^. The resulting heterogeneous microstructures often suffer reduced ductility, toughness, and fatigue life compared to the base metals^29^. FSP effectively processes the surface of the fusion weldments by transforming the microstructure, refining grains, and promoting the even distribution of particles, leading to improved mechanical properties and structural characteristics. Devireddy et al.^30^ studied the effect of FSP on the AA 2024 welded using tungsten gas arc welding (GTAW). They remarked that the FSP of the welded joints completely reduced the porosities in the welded joints and modified the microstructure and mechanical properties of welded joints after being processed. The present work is a comparative study between the effect of PWHT and TMT on the AA 6082-T6 MIG weldments. The effect of the PWHT using the T6 temper condition and the TMT using the FSP technique was carried out on the 5 mm thick AA6082-T6 MIG welded joints. Optical microscopy (OM) and scanning electron microscope (SEM) have been used to characterize the microstructure of the processed joints. The mechanical properties of hardness and tensile tests of the processed joints were studied and compared with the MIG welded joint without any treatment and the as-received materials.

## Materials and methods

### Materials

**Figure 1:**
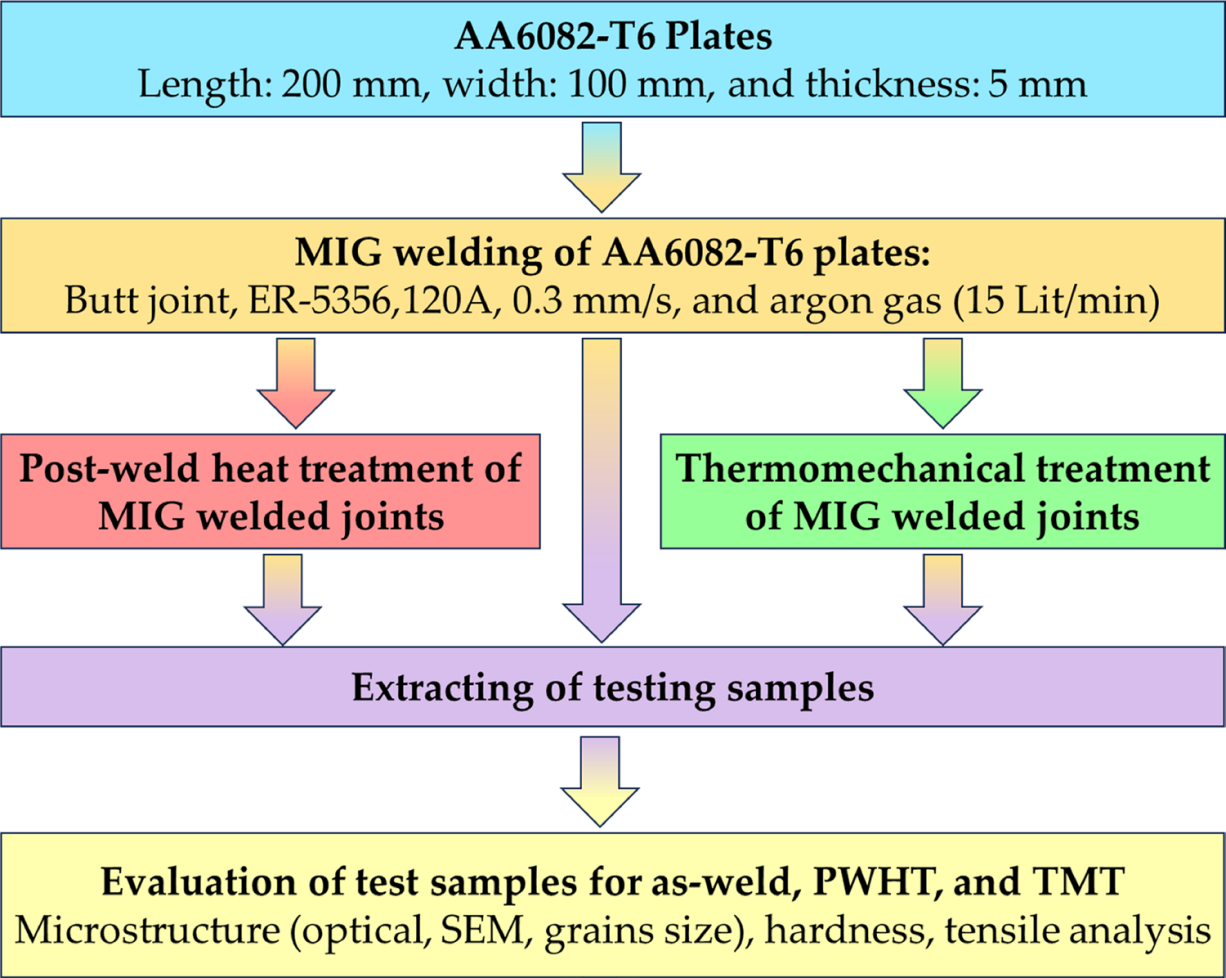
A flowchart of the conducted experimental work.

AA 6082-T6 aluminum alloy was carried as the initial material in this research with 1000 mm × 500 mm × 5 mm dimensions. The initial plates were cut to the required dimensions using a milling machine. The initial plates were resized to the necessary length of 200 mm and width of 100 mm. The nominal chemical of the AA6082-T6 plates was characterized utilizing Foundry-Master Pro spectroscopy equipment (manufactured by Oxford Instruments, Abingdon, United Kingdom). Table 1 illustrates the chemical composition of AA 6082-T6 alloy (Fig 1). A flowchart summarizes the applied methodology of the current study to processing the MIG welded joints using the PWHT and TMT techniques.

**Table 1:** Chemical composition of materials (wt. %).

Material	Cr	Cu	Fe	Mg	Mn	Si	Ti	Zn	Al
AA 6082-T6	0.25	0.10	0.52	0.83	0.62	0.88	0.10	0.20	Bal
ER 5356	0.09	0.10	0.40	5.12	0.95	0.25	0.08	0.97	Bal

### MIG welding of AA 6082-T6 alloy

**Figure 2:**
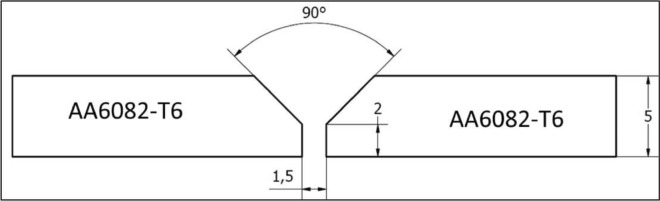
The dimensions of the V groove formed to AA6082-T6 but joint.

AA 6082-T6 joint plates were joined in similar butt joints using MIG welding machine (ESAB-EMP 210, Traverse City, USA). The welding process of AA6082-T6 plates was conducted in El Sherifein Company, Cairo, Egypt. The ER5356 welding wire with a diameter of 1.2 mm was selected to weld the AA6082-T6. Table 1 shows the composition of ER5356 welding wire according to the supplier (ESAB AB, Gothenburg, Sweden). The joint groove of V was opened to welding butt positions of plates, and the dimensions of the V groove are shown in Fig. 2. During the MIG welding process, welding current (120A), welding speed (0.3 mm/s), shielding gas (argon), and gas flow rate (15 L/min) were used. The thickness of the backing copper sheets under base metals is 1 mm. Before the MIG welding process, the surface of welded joints was ground via a stainless-steel brush to remove oxide films, and the surface was cleaned with acetone to eliminate grease or oil.

### Post-weld heat treatment of MIG welded joints

The PWHT used was the T6 temper condition. To obtain the T6 temper condition, the similar MIG welded joints were treated for 6 h at 540 °C, and then water quenched before being artificially aged for 6 h at 160 °C.

### Thermomechanical treatment of MIG welded joints

**Figure 3:**
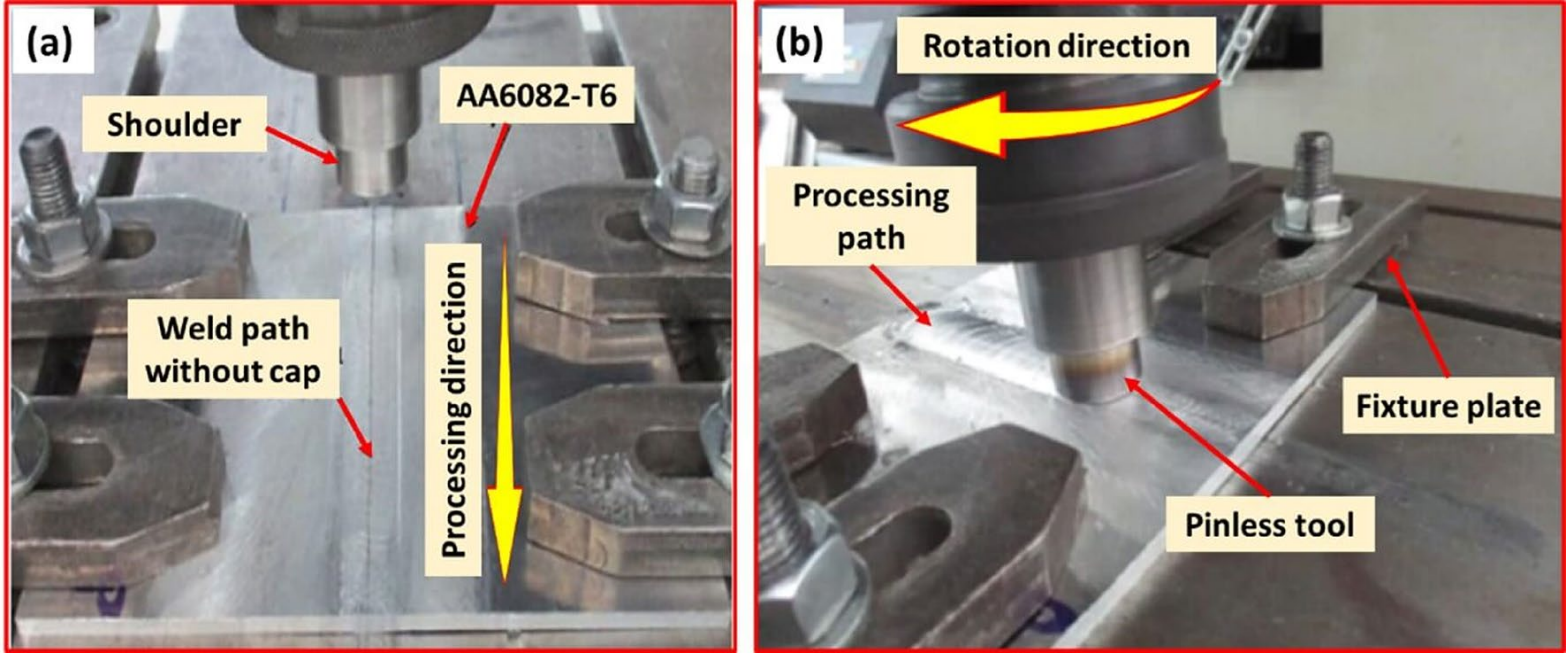
FSP of butt AA6082-T6 MIG welded joints using a pinless tool.

**Figure 4:**
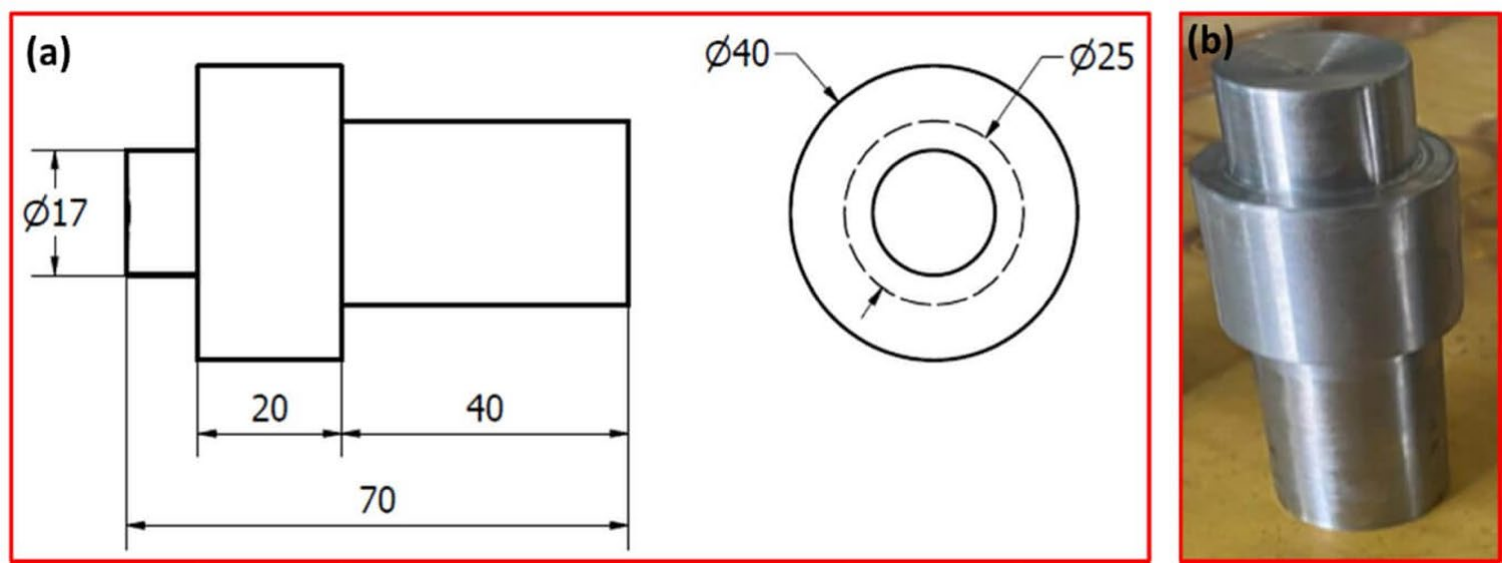
(**a**) The dimensions and (**b**) image of manufactured TMT tool.

The thermomechanical treatment (TMT) of AA 6082-T6 MIG welded joints using the FSP technique was achieved using an automatic friction stir welding and processing machine (EG-FSW-M1, Suez University, Suez, Egypt)^31,32^, as shown in Fig. 3a,b. The FSP parameters were selected after several tries to attain the optimum PSP parameters: the rotation speed was 800 rpm, the travel speed was 200 mm/min, and the tool tilt angle was 3°. The pinless tool was designed with a smooth concave shoulder, as represented in Fig. 4. It was made from W302 cold work tool steel (Bühler AG, Branch office Cairo, Egypt) and heat-treated to obtain a hardness of 52 HR.

### Characterization

**Figure 5:**
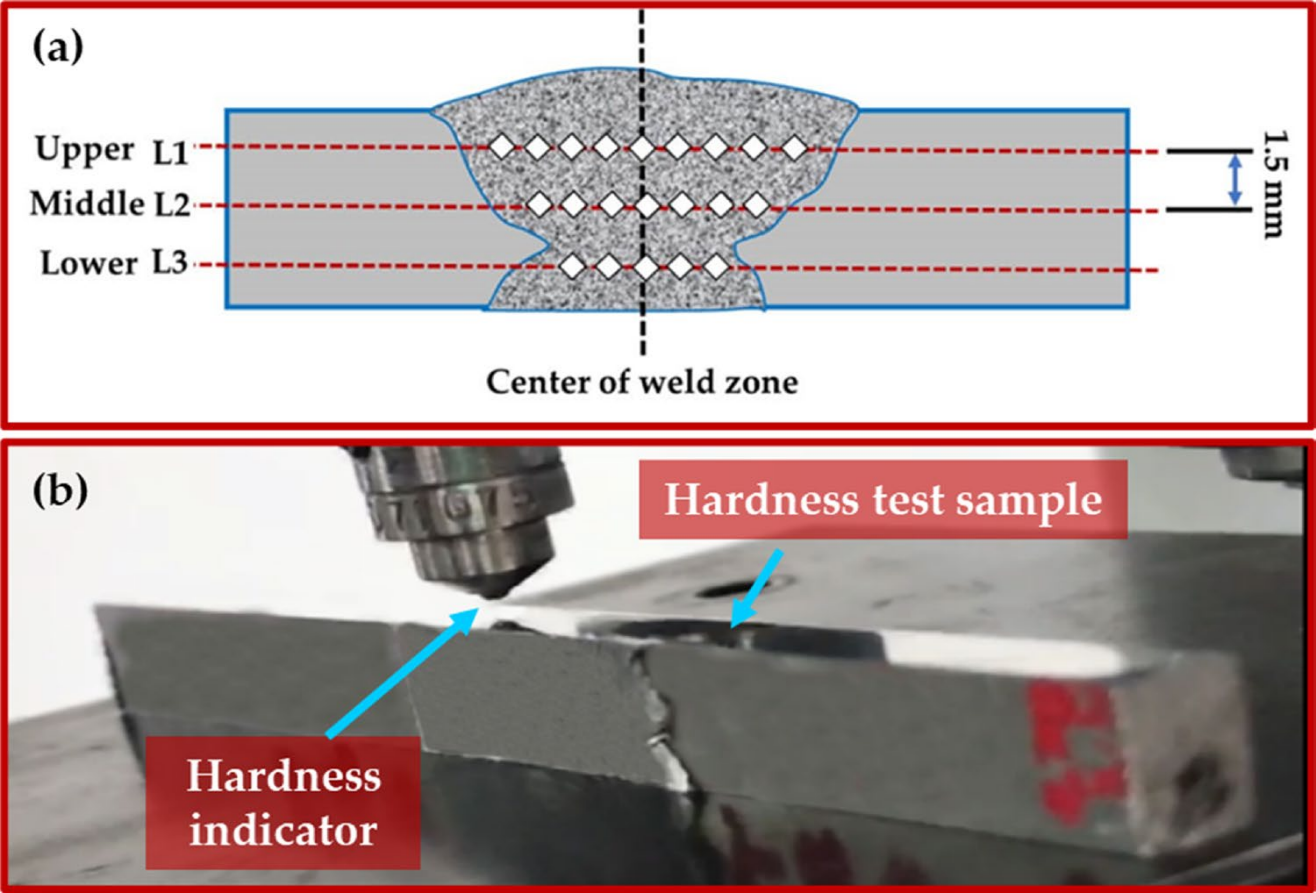
(**a**) Schematic drawing and (**b**) photo image of hardness measurements.

**Figure 6:**
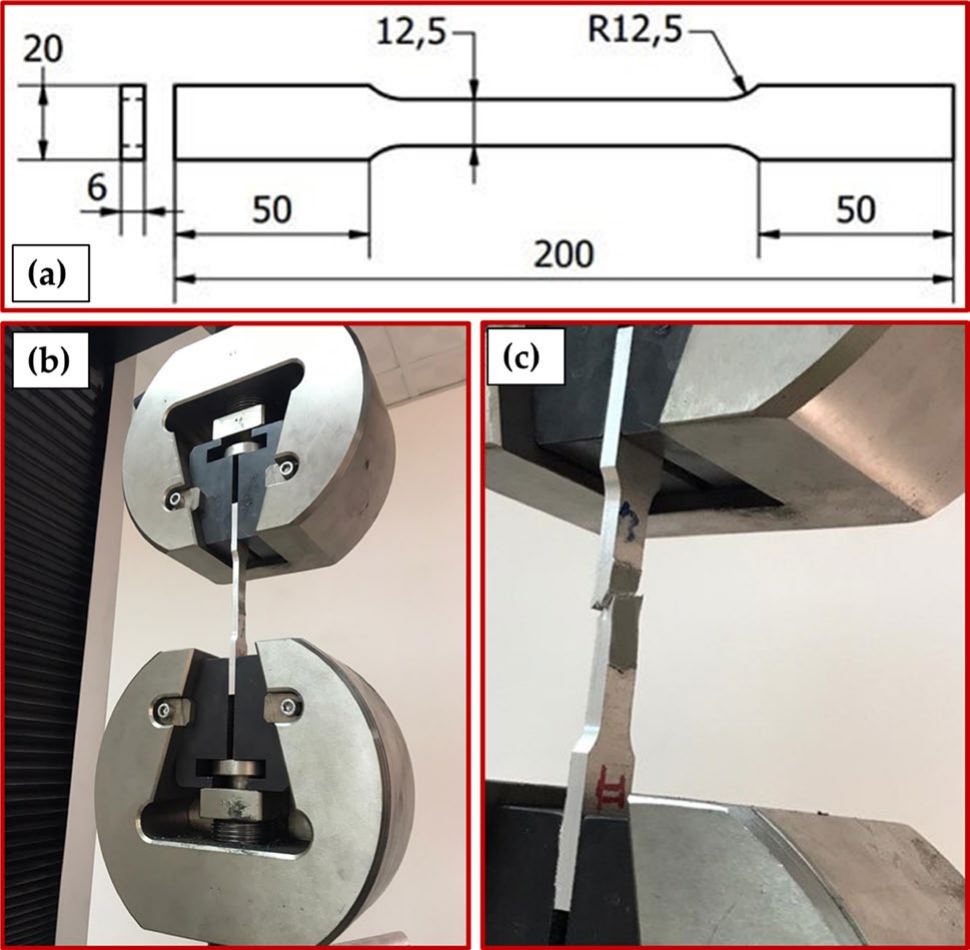
(**a**) Dimensions of tensile test specimens; all dimensions in mm. (**b**,**c**) The photo image of the tensile testing sample during and after the tensile test.

The produced samples (as-weld, PWHT, and TMT samples) were cut perpendicular to the welding path to characterize the macrostructure examination, microstructure evaluation, Vickers hardness, and tensile test. The cut samples were prepared for ground, polished, and chemically etched with Keller's regent solution standard metallurgical agents. The microstructure analysis was achieved using optical microscopy (OM, Olympus, BX41M-174 LED, Tokyo, Japan). In addition, the microstructure analysis was supported using the SEM analysis (FEG 250, FEI Company, Hillsboro, OR, USA). Vickers hardness was measured through three lines on transverse cross-sections of produced samples (Fig. 5) using a Vickers hardness tester machine (Model HWDV-75, TTS Unlimited, Osaka, Japan) under 500 g load for 15 s holding time. The tensile test specimens for all produced samples were cut along the transverse directions of the welding path according to ASTM-E8; the dimensions of the tensile test samples are represented in Fig. 6a. The tensile test was done using an Instron tensile test machine (model 4208-300 kN capacity, Norwood, MA, USA) using 0.05 mm/min machine head speed at room temperature. Figure 6b,c shows the tensile testing sample during and after the tensile test. The SEM equipped with an EDS analysis (model Quanta FEG 250, FEI Company, Hillsboro, OR, USA) was used to characterize the microstructure of the produced samples and the fracture surfaces of the tensile failed samples.

## Results and discussion

### Macro and microstructure analysis

**Figure 7:**
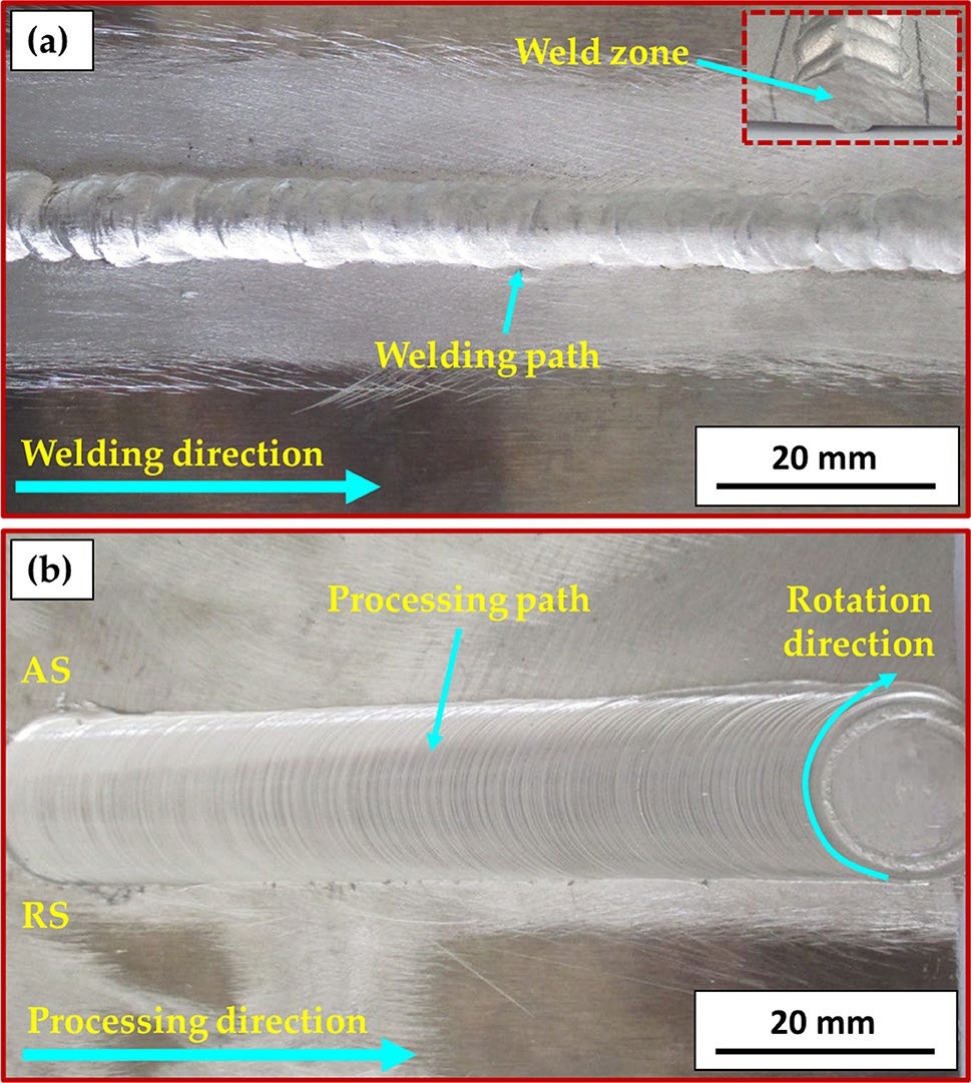
Top-view images of the 5 mm thick AA6082 MIG welded joints after (**a**) PWHT and (**b**) TMT processes.

**Figure 8:**
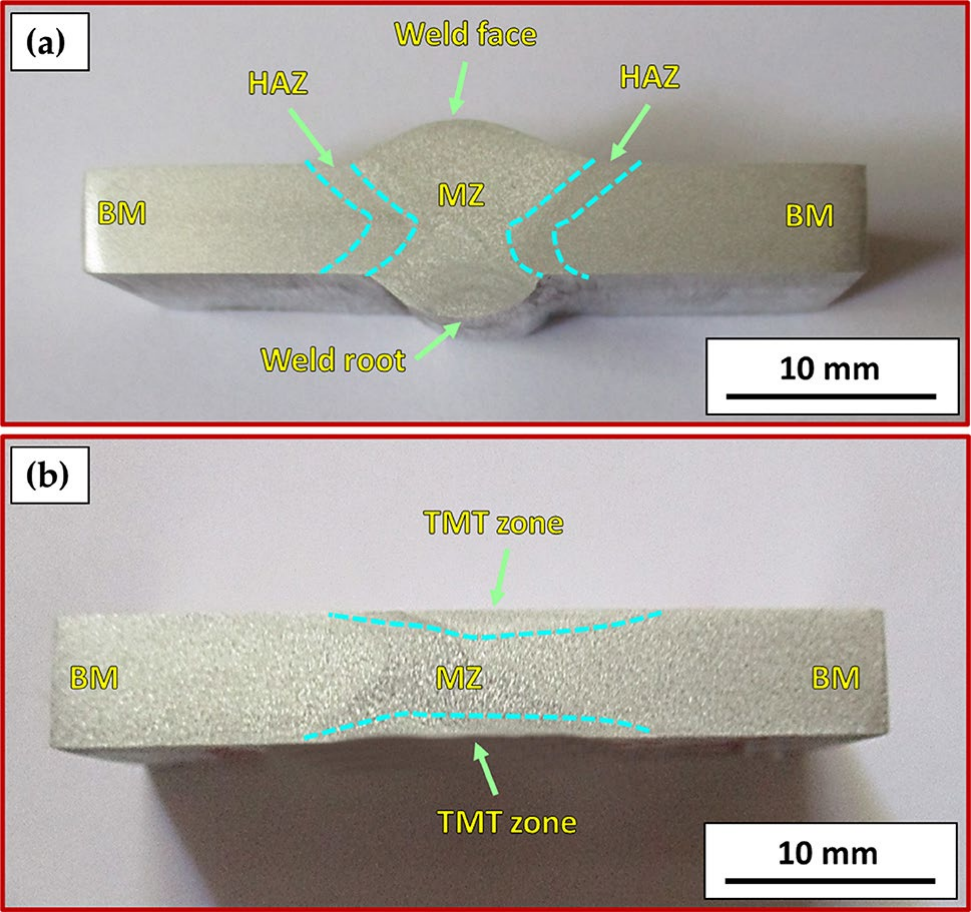
Cross-section view of the 5 mm thick AA6082 MIG welded joints after (**a**) PWHT and (**b**) TMT processes.

**Figure 9:**
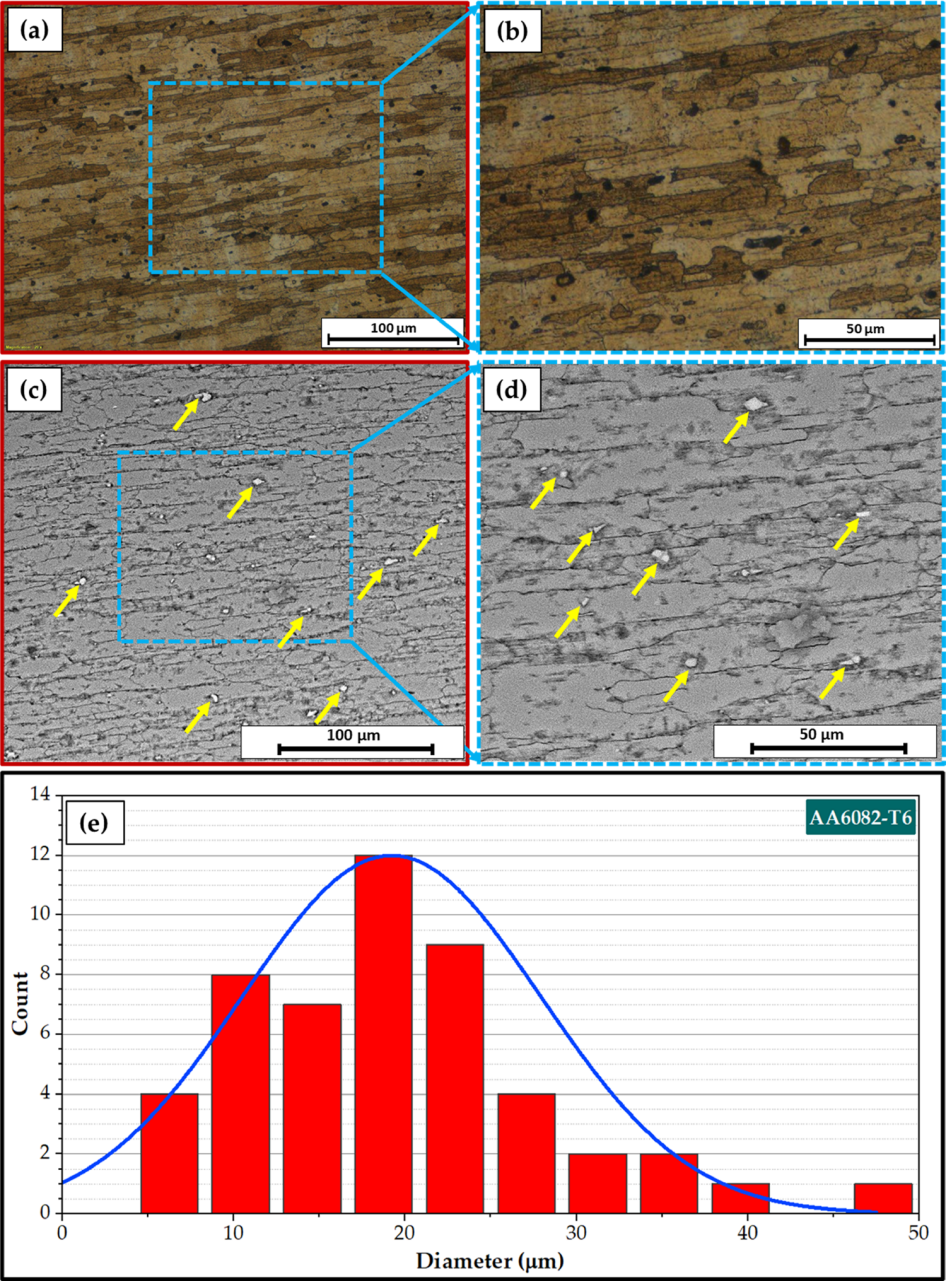
(**a**,**b**) Optical, (**c**,**d**) SEM microstructure, and (**e**) grain size analysis of the as-received AA 6082-T6.

**Figure 10:**
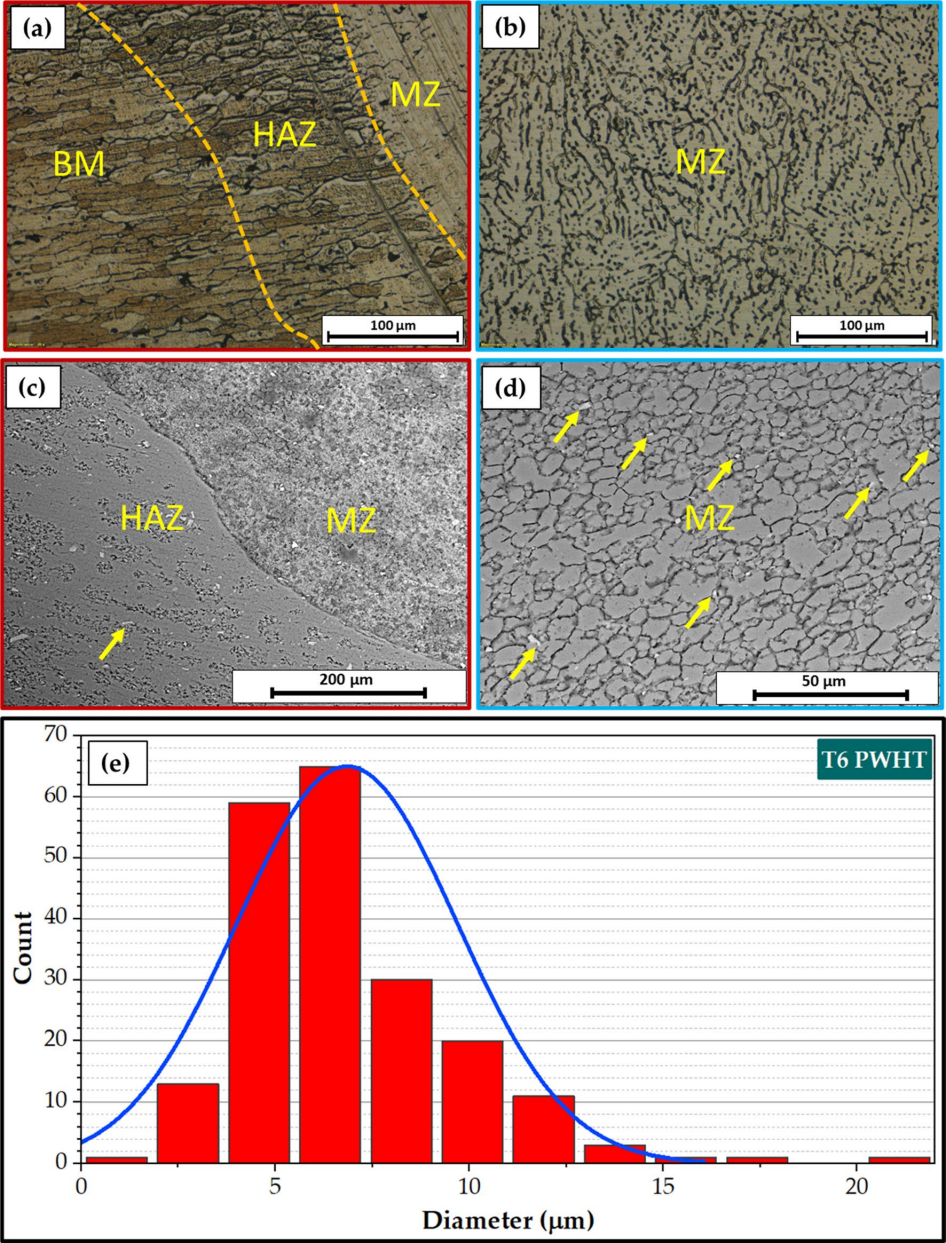
(**a**,**b**) Optical, (**c**,**d**) SEM microstructure, and (**e**) grain size analysis of the heat-treated (PWHT) AA6082-T6 MIG welded joint.

**Figure 11:**
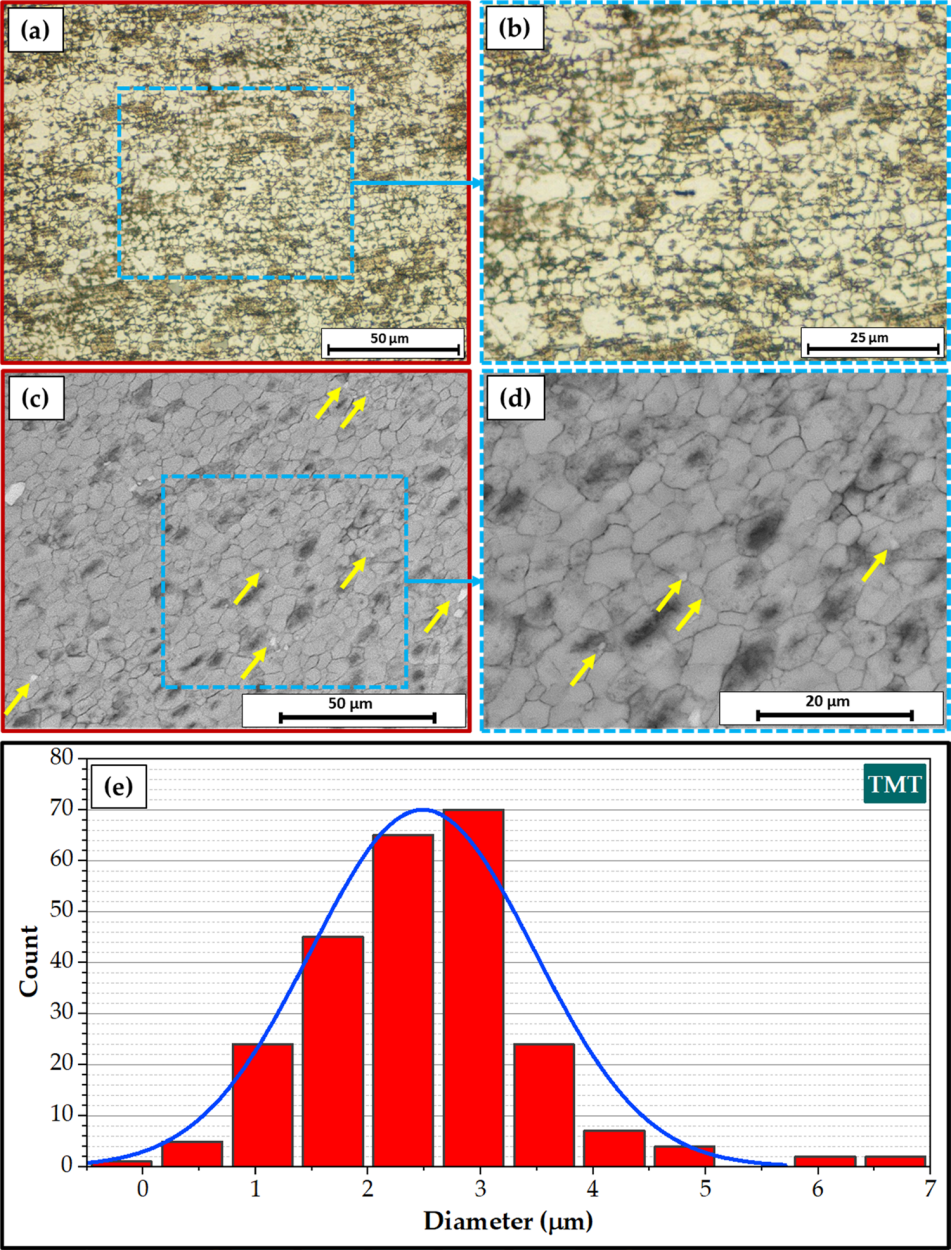
(**a**,**b**) Optical, (**c**,**d**) FE-SEM microstructure, and (**e**) grain size analysis of the TMT region of the AA 6082-T6 MIG welded joint.

Figure 7 illustrates a top view of AA6082 MIG welded joints after PWHT in the T6 temper and TMT processes. Both processed joints appear acceptable without any evident surface defects. Figure 8 presents the cross-section view of the 5 mm thick AA6082 MIG joints processed utilizing the PWHT and TMT methods. No defects, such as tunnels or cracks, were observed in the investigated cross-sections of the processed joints. Furthermore, as anticipated, the weld and heat-affected zones exhibit features characteristic of the welding technique applied. For the PWHT joint, the weld zone displayed a dumbbell shape for both MIG processes resulting from the welding procedure and joint design. Additionally, no welding defects were detected, such as substantial pores or undercuts. Similarly, no defects were apparent for the TMT joint. However, some minor observations were observed. The considerable joint reinforcement of the PWHT MIG joints ranged from 2 to 3 mm, as depicted in Fig. 8. In contrast, the thickness of the TMT joint was reduced from 5 to 4.6 mm following the friction stir processing of both the upper and lower surfaces of the processed area. A small flash was seen on both sides of the TMT path. The optical microstructure (OM), FE-SEM, and grain size analysis results of the as-received AA6082-T6 are displayed in Fig. 9. It can be remarked that the general microstructure of the OM see in Fig. 9a,b and FE-SEM see in Fig. 9c,d techniques are similar, which appeared in the elongated grains structure belonging to the rolling direction of the rolled AA 6082-T6 initial sheets. The same features of the rolled AA 6082-T6 microstructure are observed in other studies^16,33^. The 5 mm thick AA6082-T6 rolled sheet has an average grain size of 19.58 ± 3 μm, as illustrated in Fig. 9e. Figure 10a–d, depict the OM and FE-SEM of the T6 PWHT of the AA6082-T6 MIG weldment. The MIG joint of the AA6082-T6 involves the three different zones formed during the MIG process; see Fig. 7a,d. These zones include the molten zone (MZ), the heat-affected zone (HAZ), and the base material (BM). In the MZ, the arc's heat forms the molten metal and then cools to form the weld bead. The HAZ is the area surrounding the MZ that experiences thermal cycles and is subjected to high temperatures but does not reach the melting temperature. The BM is unaffected by the welding temperature outside the HAZ. The MZ has an average grain size of 7.67 ± 4 μm, as shown in Fig. 10e. The OM and FE-SEM microstructures of the TMT zone of the AA 6082-T6 MIG weldment are shown in Fig. 11a–d respectively. The TMT zone is formed on the MIG joint's upper and lower surfaces utilizing the rotating tool's stirring action, which is applied during the friction stir process. Several distinct features characterize the TMT zone. One of the most notable features is the presence of refined and equiaxed grains formed due to dynamic recrystallization. This phenomenon occurs due to the high strain and heat generated in the TMT zone, which causes the MZ coarse-grained (Fig. 10a–d) structure to transform phase. Additionally, the TMT zone (Fig. 11a–d) exhibits a high degree of grain refinement, which can result in improved mechanical properties of the treated joint. Figures 9 and 10 indicate that the MZ region has a course equiaxed grain structure compared to the formed structure during the TMT process (Fig. 11). The TMT region has a grain refining of around 83% and 55% compared to the AA 6082-T6 BM and the MZ region, as shown in Figs. 10d and 11d. The bright features (indicated by yellow arrows, Fig. 9) are constituent Al-Fe-Mn-Si-(Cr) intermetallic particles^34^, which, to an extent, are aligned along the rolling direction. After the PWHT, Al-Fe-Si phases are more stable and persist after the heat treatment process^35^, as shown in Fig. 10. During the TMT process, the intense plastic deformation and material flow induced lead to fragmentation and refinement of intermetallic (Fig. 11) particles present after welding^11,32^.

### Mechanical properties

**Figure 12:**
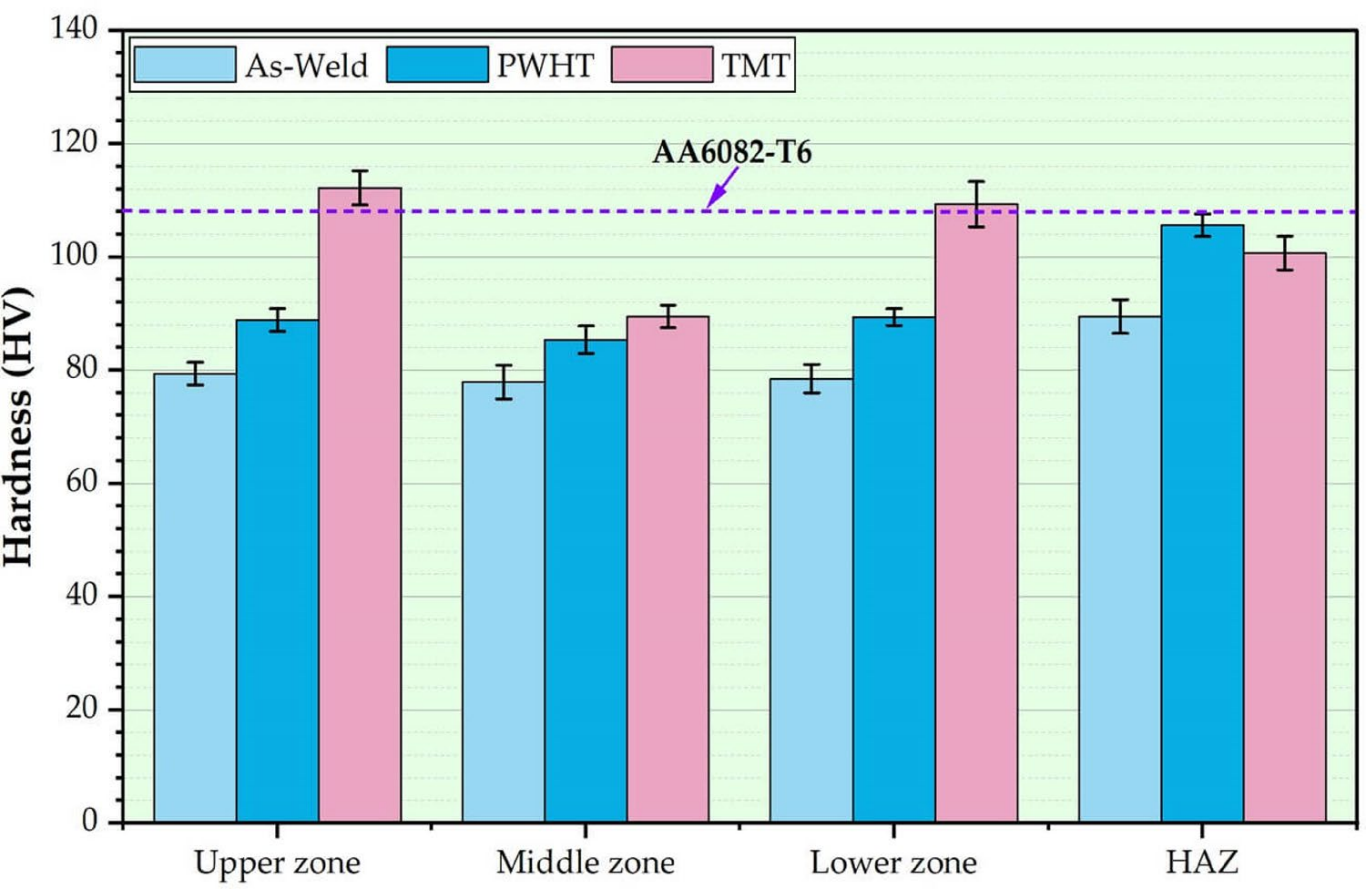
Hardness measurements of the as-weld, PWHT, and TMT joints.

Figure 12 shows the hardness of the AA 6082-T6 as-weld MIG joints and the treated joints using the PWHT and TMT techniques. The hardness measurements of the as-weld, PWHT, and TMT specimens were done at three levels of the cross-section of the specimens, as illustrated in Fig. 5. The average hardness results in the different zones of upper, middle, lower, and HAZ are shown in Fig. 12. Firstly, for the as-weld joint, the hardness measurements indicated a reduction in the hardness value of the MZ of the as-weld joints compared to the AA 6082-T6 initial material. The decrease in microhardness in the MZ was attributed to two main responses. The first reason was that the microhardness of the used ER 5356 filler rod was lower than that of the AA 6082-T6 initial joint plates. The second reason was the loss of alloying elements due to the melting process during the MIG welding. Han et al.^36^ studied the effect of the laser MIG hybrid welding process on the 6 mm thick AA 6082-T6. They reported that the hardness of the MZ of all welded joints is lower than that of the base material due to higher heat input during the welding process, which leads to the loss of strengthening alloying elements and dissolves the strengthening phases in the MZ. In addition, Wang et al.^37^ also reported that the low hardness values in the MA during the hybrid laser MIG welding process of A 6N01S-T5 aluminum alloy are due to the resolved major strengthening phase of *β*^*'*^ during the thermal cycle of the applied welding process. Secondly, for the PWHT process of the MIG welded joints, the hardness value of the as-welded joint was 79.4, 77.9 HV, and 78.5 HV for the upper, middle, and lower zones, respectively, on the cross-section of the MIG welded joint, as shown in Fig. 12. By executing the PWHT for the MIG welded AA 6082-T6, the hardness value was improved by 12%, 10%, and 15% for the upper, middle, and lower zone respectively, compared with the as-welded joint. This could be described by the uniform and fine distribution of precipitates at the welded joint treated by applying PWHT^38^. The improvement in hardness for the electron beam welding joints by using the PWHT compared to the as-weld joint of AA 2219 alloy was reported by Malarvizhi et al.^39^, and they found that the MZ of the joint treated by the PWHT consists of a uniform distribution and very fine precipitates compared with other welded joints. This was selected as a main contributor to improving the hardness values for the PWHT joints. Thirdly, for applying the TMT on the upper and lower surface of the AA 6082-T6 MIG welded joint, the hardness was remarkably improved by 24% and 23% for the upper and lower zone, respectively, compared with the PWHT joint. And over 38% and 37% for the upper and lower zones, respectively, compared to the as-weld joint. This improvement in the hardness properties of the joints treated by the TMT process is due to the recrystallization and grain refinement^40^. During the TMT process, the tool rotates along the MIG welded joint's surface, creating a plasticized zone characterized by high strain rates and temperatures. This results in a reduction in the grain size in the processed zone and an increase in the number of sub-grains, which increases the hardness of the processed materials in the upper and lower surfaces of the treated joint^41,42^. So, on the first hand, there was no remarkable change in the hardness values through the welded joint thickness of the as-weld and PWHT joints (Fig. 12). On the other hand, the hardness of the MIG welded joint treated by the TMT process is higher at the upper and lower surface than the middle zone hardness. This variation in hardness is due to the pinless tool utilized to treat the surface of the MIG welded joint using FSP technology. Furthermore, the average hardness measurements of the as-weld, PWHT, and TMT joints," the average hardness of the middle zone for the PWHT and the TMT treated samples observed unnoticeable improvement due to overlap limits of the error bar. That was the result of the TMT process being applied only on the upper and lower surface of the MIG joint using the FSP pinless tool.

**Figure 13:**
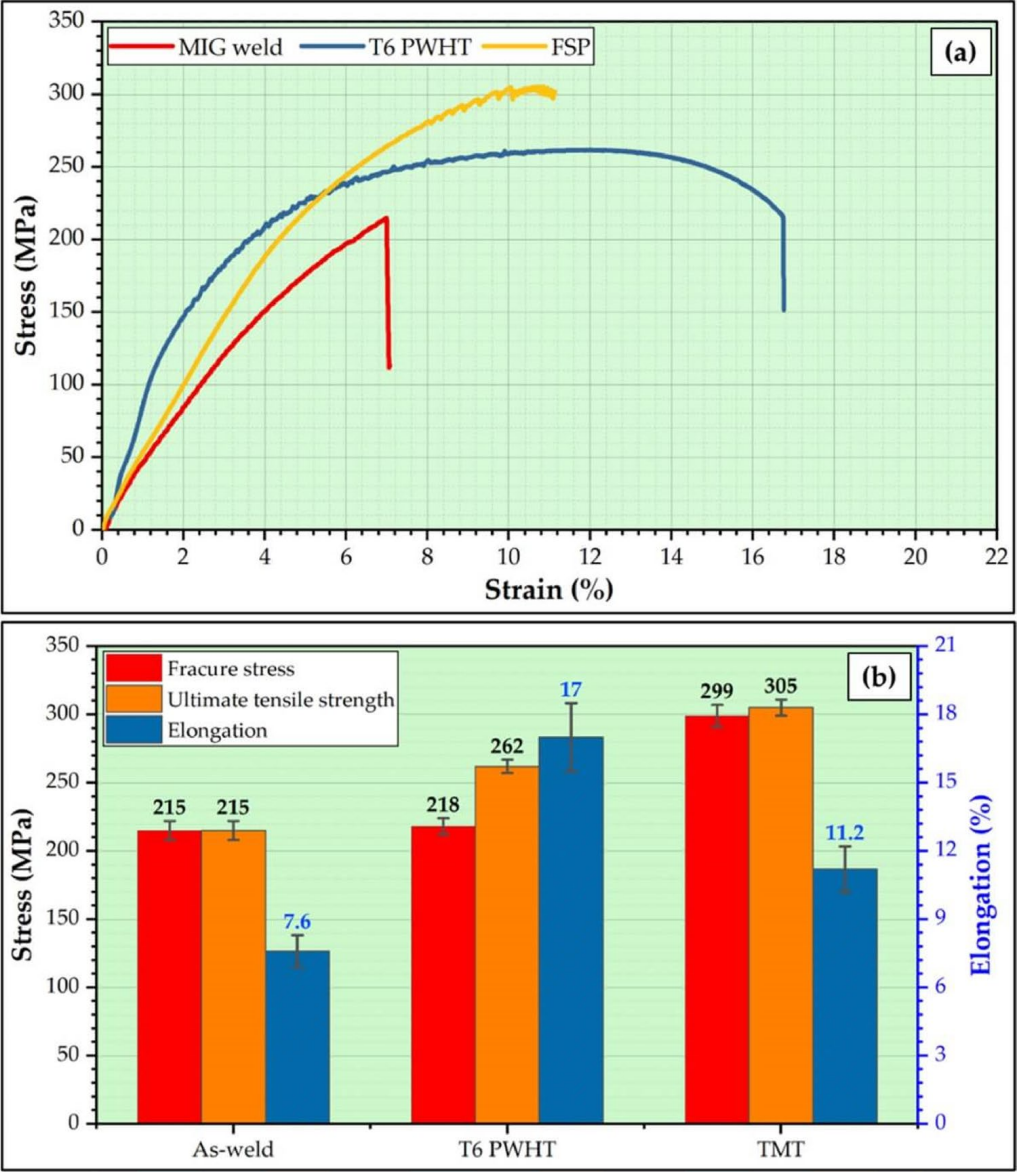
(**a**) Stress–strain curves and (**b**) tensile properties of the as-weld and processed joints using the PWHT and the TMT procedures.

Figure 13 shows the tensile stress–strain curve (Fig. 13a) and the tensile properties (Fig. 13b) of the as-weld MIG joint and the treated joints using the PWHT (T6 temper condition) and TMT (surface treatment) processes. The as-welded joint was recorded to have fractured on the advanced side in the HAZ (Fig. 13a). Due to the HAZ being the location where there is no strain throughout the thermal cycles, this behavior is expected. In addition, the HAZ has the lowest tensile strength compared to the MZ and the parent materials^37,43^. The MIG welded joint treated by the PWHT was noted to have fractured at the MZ (Fig. 13a). Wang et al.^43^ studied the effect of PWHT on the properties of the AA 6082-T6 MIG welded joints. They found the tensile location of the treaded joints by the PWHT occurred in the MZ, and they attributed it to the Mg_2_Si precipitates being dissolved into the matrix during the PWHT process; then, the fine precipitates of Mg_2_Si were homogeneously distributed, resulting in the HAZ properties was improved.

Furthermore, in the PEREZ et al.^44^ investigations, the PWHT forms the Mg_2_Si precipitates and can be transformed to the *β"* during artificial aging (T6), resulting in recovered properties of the AA 6061-T6 welded joint to the parent materials. The MIG welded joint treated by the MTM technique was fractured in the BM zone away from the MZ and HAZ (Fig. 13a). During the TMT process, a rotating pinless tool is utilized to stir the material at the upper and lower surface of the MZ. This process can help break up any coarse grain structures (Fig. 10) that may have formed during the MIG welding process, leading to a more refined microstructure (Fig. 11) of the TMT zone^45,46^. Therefore, the properties of MZ and HAZ were improved at the upper and lower surfaces of the MIG welded joints. The welded joint treated by the PWHT (T6 temper condition) revealed a UTS of 262 MPa and a tensile strain of 17%, with fracture stress of 218 MPa. The PWHT joint is higher than the as-weld MIG joint, with a UTS of 56.12 MPa, a tensile strain of 7.6%, and a fracture stress of 215 MPa (Fig. 13b). Furthermore, the MIG welded joint treated by the TMT process exhibited higher UTS than the as-weld and PWHT joints with 305 MPa of UTS, 11.2 tensile strain, and 299 MPa of fracture stress (Fig. 13b). Table 2 illustrates the joint efficiencies and the strain-hardening capacities of the as-weld, PWHT, and TMT joints. It can be noted that the TMT-treated joint has a higher joint efficiency of 99% compared to 69.8 and 85.1 for the as-weld joint and the joint treated by the PWHT, respectively. The strain-hardening capacity of the welded and treated joints is listed in Table 2. A welded joint's strain-hardening capacity (SHC) describes the ability to resist further plastic deformation after yield stress. If the value of the SHC of the welded joint is low, the joint may be more prone to deformation or failure under lower stresses.

**Table 2:** The joint efficiency and the strain-hardening capacity of the welded and processed joints.

Treatment process	Joint efficiency (%)	Strain hardening capacity
AA 6082-T6	-	0.14
As-weld	69.8	0.556
T6 PWHT	85.1	0.690
TMT	99.0	0.704

On the other hand, if the value of the SHC of the welded joint is high, it will be more resistant to deformation and failure under stress. Table 2 shows that the MIG welded joint treated by the TMT process has a higher SHC of 0,704 compared to the as-weld (0.556) and the MIG joint treated by the PWHT (0.690). These results refer to the MIG welded joint treated by the TMT, which is preferred for applications where the welded joint is subjected to high loads or cyclic loading, as they are more resistant to deformation and failure than other produced joints.

**Figure 14:**
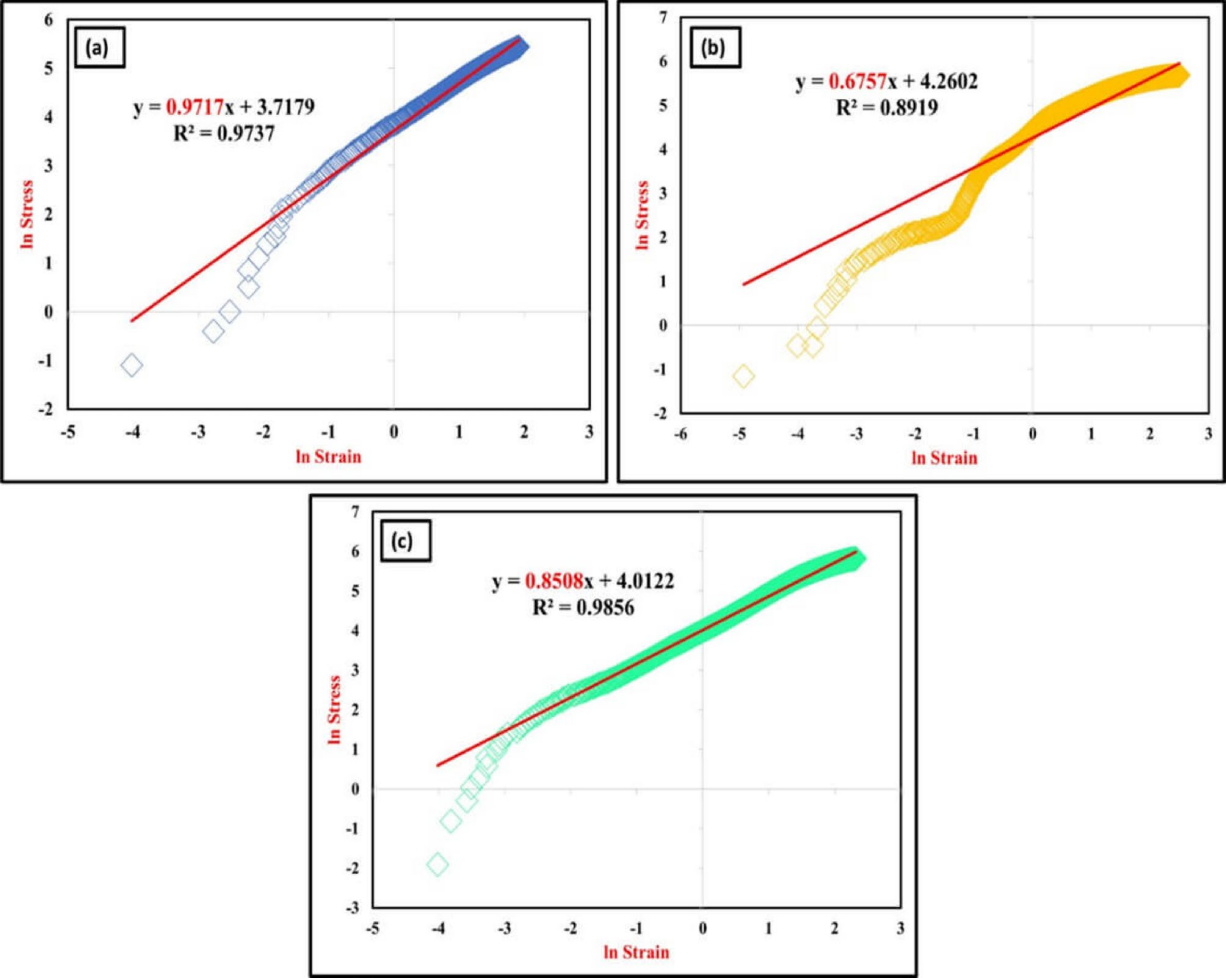
Strain-hardening behavior/working hardening of the (**a**) as-weld, (**b**) PWHT and (**c**) TMT joints.

After the tensile test, the strain-hardening behavior/working hardening for welded samples by MIG welding, T6 PWHT, and FSP was calculated using Excel Software to analyze the stress–strain results, as shown in Fig. 14. The tensile flow behavior's noteworthy aspect is the strain-hardening coefficient for the welded joints. Consequently, the welded joints created by MIG welding had the highest strain-hardening coefficient (0.9717) compared to the welded joints created by T6 PWHT and FSP, whereas the welded joints created by T6 PWHT had the lowest values.

**Figure 15:**
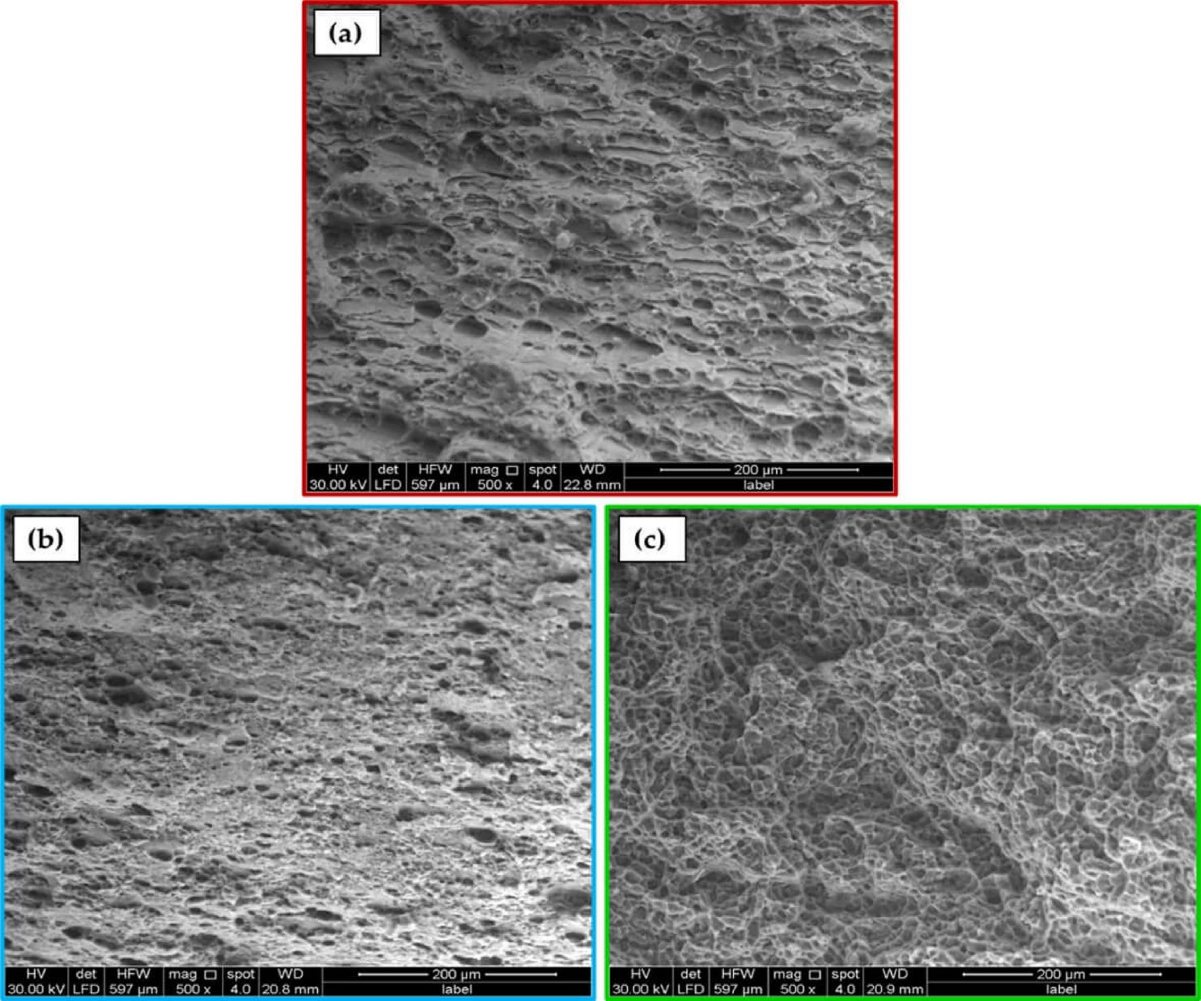
The FE-SEM images of the fracture surface of the (**a**) as-weld MIG joint, processed joint using the (**b**) PWHT and (**c**) TMT joints.

The fractography analysis of the AA 6082 MIG welded joint (Fig. 15a), the PWHT joint (Fig. 15b), and TMT joint (Fig. 15c) were conducted using an FE-SEM, as illustrated in Fig. 15. The FE-SEM analysis of the MIG welded joint revealed the presence of large and shallow dimples accompanied by areas of plastic deformation. These observations suggest that the MIG welded joint exhibited a ductile failure mechanism. Following the implementation of treatment methods such as PWHT and TMT, discernible disparities can be observed in the morphology and dimensions of dimples and other fracture characteristics. Following the PWHT of the MIG welded joint, the fractographic analysis reveals the presence of both big and small deep dimples accompanied by cleavages and serrations. These observations indicate the occurrence of mixed fractography modes, although the predominant fracture mode is brittle. Following the TMT procedure, it was observed that the dimples feature exhibited deep and shallow fine dimples only in the fractured region of the TMT joint. This finding perhaps explains the superior tensile capabilities of the TMT-processed joint in comparison to both the MIG as-welded joint and the joint processed using the PWHT procedure (Fig. 13).

## Conclusions

This study evaluates the effect of post-weld heat treatment (PWHT) and thermo-mechanical treatment (TMT) techniques on the microstructure and mechanical properties of AA 6082 MIG welded joints. Based on the results obtained, it can be inferred that the following conclusions can be drawn:

1. The PWHT and the TMT's applied treatment regimes succeeded in modifying the microstructure and improving the mechanical properties of the AA 6082 MIG weldments.
2. The MIG joint using the TMT procedure revealed grain refining of around 83% and 55% compared to the AA 6082-T6 initial plate.
3. In the TMT processed zone, the hardness remarkably improved by 24% and 23% for the upper and lower zones, respectively, compared with the PWHT joint, and over 38% and 37% for the upper and lower zones, respectively, compared to the as-weld MIG joint.
4. The MIG welded joint treated by the TMT process exhibited higher UTS than the as-weld and PWHT joints with 305 MPa of UTS, 11.2 tensile strain, 299 MPa of fracture stress, and joint efficiency of around 99%.

## Data Availability

The datasets used and/or analyzed during the current study available from the corresponding author on reasonable request.
